# Can recurrences be predicted in craniopharyngiomas? β-catenin coexisting with stem cells markers and p-ATM in a clinicopathologic study of 45cases

**DOI:** 10.1186/s13046-017-0562-9

**Published:** 2017-07-14

**Authors:** Elia Guadagno, Oreste de Divitiis, Domenico Solari, Giorgio Borrelli, Umberto Marcello Bracale, Alberto Di Somma, Paolo Cappabianca, Marialaura Del Basso De Caro

**Affiliations:** 1 Department of Advanced Biomedical Sciences, Pathology Section, University of Naples Federico II, Via Pansini 5, 80131 Naples, Italy; 2 Department of Neurosciences, Reproductive and Odontostomatological Sciences, Division of Neurosurgery, University of Naples Federico II, Via Pansini 5, 80131 Naples, Italy; 3 Department of Public Health, Unit of Vascular and Endovascular Surgery, University of Naples Federico II, Via Pansini 5, 80131 Naples, Italy

**Keywords:** Craniopharyngiomas, β-catenin, Recurrence, CD166

## Abstract

**Background:**

Recurrence is a common feature of craniopharyngiomas, benign tumors that origin from squamous epithelial remnants of Rathke’s pouch- arising at any segment of its whole course. There are two histotypes, showing different morphology and clinical behavior: adamantinomatous(adaCP) and papillary (papCP). An univocal strategy of management has not yet been defined, being considered the combination of surgery and radiotherapy the most effective, especially in case of incomplete resection. Therefore, the identification of factors influencing the biological and clinical behaviour is of paramount importance.

β-catenin is a cell-cell adhesion protein, whose nuclear localization has been linked to the pathogenesis of adaCP: its nuclear accumulation is associated to the presence of a tumor stem cell subpopulation. The latter is made of cells capable of self-renewal, hence believed to be responsible of recurrence, metastases and resistance to therapy in all tumors.

ATM is a kinase activated by autophosphorylation (p-ATM) upon DNA double-strand breaks. It is involved not only in DNA repair, but also in tumor migration and invasiveness. Its expression may have prognostic implications in many neoplastic diseases.

**Methods:**

In this study, we measured the immunohistochemical expression of β-catenin, stem cell markers (CD133, CD166), Ki67 and pATMin 45 craniopharyngiomas and correlated it with clinicopathologic features.

**Results:**

Statistical analysis revealed strong correlation of β-catenin with recurrence (*p* = 0.0039), Ki67 (*p* = 0.0011, *r* = 0.4903) and CD166 (*p* = 0.0002, *r* = 0.6218). A slight tendency to a higher expression of β-catenin was recorded for adaCP rather than papCP (*p* = 0.0895).Fisher’s exact test showed that CD166 was significantlyrelated with recurrence (*p* = 0.0040). Furthermore, cytoplasmic pATM was more expressed in adaCPs (*p* = 0.0470), compared to papCPs that displayed a more evident nuclear signal (*p* = 0.0313) instead.

**Conclusions:**

Backing upon these data, we could weigh in on the need of identifying β-catenin and CD166 as prognostic markersthat could be useful in predicting thebiologicalbehavior, as recurrence risk incraniopharyngiomas. The final goal is to drew up a prognostic algorithm to be of aid in the planning of an appropriate treatment strategy. Furthermore, our findings demonstrate that pATM could be used as additional distinction-marker between the two histotypes.

## Background

Craniopharyngiomas (CPs) are rare benign tumors of the sellar and parasellar region, classified as low histologic grade (WHO grade I) that account for 2–4% of all intracranial tumors [1]. Two histological subtypes exist, namely adamantinomatous (adaCP) and papillary (papCP), differing for age distribution, frequency, biology and clinical outcome. The former are more frequent, with two peaks of incidence (under 15 years and between 50 and 74 years), and a heavy tendency to infiltrate the surrounding structures (hypothalamic/pituitary axes and optic nerve/chiasma), by finger-like protrusions. On the other side, papCPs are less common and mainly affect adults.

Thus far, the possibility of identifying inner tumor factors influencing the biological and clinical behavior of this “chronic disease” is paramount.

Pathogenesis of CP accounts on an embryogenic theory for adaCP and a so-called metaplastic for papCP: the adamantinous type arises from remnant cells of the Rathke’s pouch, while the latter from adenohypophyseal cells, which underwent to squamous metaplastic changes [2, 3]. Dysregulation of the WNT/β-catenin signaling pathway is involved in the pathogenesis of adaCP: hence the presence of cells that accumulate β-catenin in the nucleus and cytoplasm and that gather along to form whirl-like structures can be detected. Activating mutations of the beta-catenin gene (exon3 of CTNNB1 gene) can be identified in the majority of adaCPs [4, 5], but not in papCPs, nor in other tumors of the sellar region [6]; the vast majority of papCPs harbor the oncogenic BRAF V600E mutation: CTNNB1 and BRAF alterations are mutually exclusive, clonal and specific to each subtype [7, 8].

In case of exon3 deletion of CTNNB1 gene, an active form of β-catenin protein is translated but it lacks the amino acids that allow its phosphorylation and then its degradation, thus resulting in an over-activation of the WNT/β-catenin pathway [9].

Studies in mice showed that WNT/β-catenin pathway is required for normal pituitary morphogenesis and differentiation. A constitutive activation of this pathway in Rathke’s pouch undifferentiated precursor cells is at the base of childhood-onset adaCP, while CTNNB1 mutations in β-catenin accumulating cell clusters are the cause of adulthood-onset adaCP [10]. Growing evidences showed that cells forming clusters are Sox2+ stem cells and that stem cell marker CD133 is co-expressed with β-catenin in adaCPs [11]. Furthermore, cells showing β-catenin accumulation displayed low proliferative activity (Ki67).

Complete removal at first surgical attempt has been suggested as the most effective treatment, although itcould eventually determine visual impairment, endocrinological disturbances, and/or hypothalamic disturbances, resulting in impairment of social and behavioral disturbances. However, craniopharyngiomas can recur even after radical resection with a rate of 23% [2] and, whether subtotal resection has been achieved, the incidence of recurrence is higher [12–16]. The treatment of the primary tumor and of recurrences with different integrated therapeutic approaches may be necessary in order to achieve long-termand, eventually, preserve a good quality of life.

When analyzing the issue of recurrence, the need of defining morphologic (large size, adhesiveness to surrounding vascular or neural structures, tumor consistency), histologic (adamantinomatoushistotype, presence of β-catenin + cell clusters, peritumoral glial reaction, brain invasion) and/or molecular factors (Ki67 and p53) is crucial to rule out the predictive role [17], but reliable factors are still lacking.

Stem cells represent a small proportion of tumor cells capable of self-renewal and differentiation towards mature cells, hence believed to be responsible of cancer evolution, recurrence, metastases and resistance to chemotherapy and radiation in all tumors [18–21]. CD133, a glycosylated five-transmembrane protein, and CD166, an activated leukocyte cell-adhesion molecule, represent cancer stem cell markers with a negative prognostic role in many solid tumors [22].

ATM is a kinase activated by autophosphorylation (p-ATM) upon DNA double-strand breaks arising from errors during replication, byproducts of metabolism, chemotherapy or ionizing radiations [23]. ATM expression has been evaluated in many tumor types, finding a different prognostic role in each of them.

The aim of our study was to assess the expression of β-catenin and compare it with stem cell markers, as CD133 and CD166, with the proliferative index (Ki67 Labeling index) in a quite conspicuous number of craniopharyngiomas. Furthermore, we measured pATM expression in all those cases. The results were then compared with the recurrence rate, in order to evaluate the potential prognostic role of all these factors.

## Methods

### Collective

Forty five samples of craniopharyngioma were analyzed. They were all obtained as formalin fixed tissue from the archive of the Department of Anatomic Pathology of the Federico II University Hospital of Naples. Patients underwent surgery, with endoscopic endonasaltransphenoidalapproach, at the Department of Neurosurgery of the same hospital. The cases were identified between 1998 and 2015 and the diagnoses were made in accordance with the World Health Organization (WHO) classification (1). Only specimens containing sufficient amountsof the respective CP subtype were taken into account. Of each specimen, samples exclusively made of fibro-inflammatory reaction, that is common in this type of pathology, were excluded from this analysis.

For each patient, follow-up data were available and we had a written informed consent to use part of the specimen for scientific and/or research scopes.

### Immunohistochemistry

Four μm sections were used for immunohistochemistry. Sections were dewaxed in xylene, hydrated in graded series of alcohol and subjected to heat-induced antigen retrieval(10 mM Sodium Citrate, 0.05% Tween 20, pH 6.0). After blocking endogenous peroxidase activity, the tissue was incubated with monoclonal antibodies for anti β-catenin (clone 14, mouse; Roche Ventana, 1:100 dilution), CD166 (clone MOG/07, ab49496, mouse; Abcam, 1:100 dilution), p-ATM (phosphoS1981)(clone ab81292;abcam; 1: 200 dilution) and Ki67 (Clone MIB1, mouse; Dako, 1:100 dilution) and with the polyclonal antibody CD133 (rabbit; Abcam, 1:100 dilution), all for 90 min. Subsequently, the slices were rinsed and incubated with the biotinylated secondary antibody, at room temperature, for 30 min. The bound antibody complexes were stained for 3–5 min or until appropriate for microscopic examination with diaminobenzidine, and they were then counterstained with hematoxylin (30s) and mounted. Appropriate positive controls were chosen: mammary fibromatosis for β-catenin, normal skin for CD166 and CD133 and normal pancreas for p-ATM. Negative control was obtained by omitting the primary antibody.

### Scoring

All the slices were reviewed by two experienced pathologists (MD, EG), using light microscopy. In discordant cases the slides were re-evaluated on a multi-headed microscope to achieve consensus.

β-catenin was considered as positive only in case of nuclear staining and a total immunostaining score (IS) was calculated as the product of a Proportion score (**0** = 0%, **1** < 10%, **2** = 10–50%, **3** = 51–80%, **4** > 80%) and an Intensity score (**0** = no signal, **1** = weak signal, **2** = moderate, **3** = strong). The same IS was used also for CD133 and CD166 membrane signal. The 3 markers were then divided into two scoring groups, on the base of the IS: absent/low expression (IS = 0–2) and moderate/high expression (IS = 3–12).

For pATM, a different scoring system was adopted because no differences in intensity were noticed. It was based on the proportion of neoplastic cells showing a signal and on its cellular localization (C, cytoplasmic or N, nuclear): **0** = absent, **1** = <10%, **2** = 10–30%, **3** > 30%.

For the evaluation of the proliferating index Ki67 (Labeling Index, L.I.), “hot spot” areas were chosen and an average of the values on 5 adjacent fields (at least 500 neoplastic cells) was calculated: normally highly proliferating areas were excluded, namely basal cells. A cut-off of 5% was identified because the median value was of 6%.

In some cases not all the markers could be tested because of tissue exhaustion.

### Statistical evaluation

The study of association between the scoring group of each marker (absent/low or moderate/high expression for β-catenin, CD133 and CD166, and ≤5% or >5% for Ki67) and clinico-pathological features (age, gender, recurrence and histotype) was carried out by Fisher’s exact test. For pATM the population was divided into positive and negative cases.

Spearman correlation test was used to examine the correlation between β-catenin and, respectively, CD133, CD166 and Ki67 and between CD166 and Ki67.

A *p* value ≤ 0.05 was considered statistically significant. All tests were two sided and carried out with GraphPad Prism 5 software (GraphPad Software, La Jolla, CA, USA).

## Results

### Clinicopathologic features

Upon 45 patients (Table 1) 30 were males and 15 were females with mean age of 43 years (ranging from 2 to 77 years). Patients had been treated by surgical removal via endoscopic endonasaltransphenoidalsurgerythat wascomplete in 23 cases and subtotal in 22 cases; these latter received radiotherapy. In 38 cases the diagnosis was of adaCP and 7 cases werepapCP. Of 4 recurrent cases only the primary tumor tissue was included for statistical evaluation; in 9 cases only the tissue of the recurrence was available (8 adaCP and 1 papCP). Therefore 13 cases were considered recurrent tumors and 32 not.

**Table 1:** Clinicopathologic data

	Histotype^b^	Gender	Age	β-catenin^a^	CD133^a^	CD166^a^	Ki67	NpATM	CpATM	RT
1	adaprim	M	51	2 (1 × 2)	0	1(1 × 1)	2%	0	2	no
2	ada	M	61	0	0	0	3%	1	2	no
3	ada	F	13	1 (1 × 1)	1(1 × 1)	2(1 × 2)	2%	2	3	no
4	ada	F	2	2(1 × 2)	0	1(1 × 1)	10%	0	3	yes
5	ada	F	66	0	1(1 × 1)	0	2%	0	1	no
6	ada	M	9	0	0	0	1%	0	0	yes
7	adarec	M	9	9(3 × 3)	4(2 × 2)	4(2 × 2)	25%	1	0	yes
8	adarec	F	4	2(2 × 1)	3(3 × 1)	3(3 × 1)	--	0	2	yes
9	ada	F	15	1(1 × 1)	1(1 × 1)	1(1 × 1)	5%	1	1	no
10	paprec	M	58	0	0	2(1 × 2)	6%	2	2	no
11	adarec	M	63	6(2 × 3)	2(2 × 1)	6(3 × 2)	15%	0	2	yes
12	adarec	M	51	2(1 × 2)	4(2 × 2)	4(2 × 2)	5%	0	1	yes
13	pap	F	47	0	6(3 × 2)	4(2 × 2)	6%	3	0	yes
14	pap	M	57	0	4(2 × 2)	4(2 × 2)	5%	3	0	no
15	adaprim	M	67	4 (2 × 2)	3(3 × 1)	3(3 × 1)	10%	0	0	yes
16	ada	M	48	0	0	0	2%	0	1	yes
17	adarec	M	66	4(2 × 2)	2(2 × 1)	3(3 × 1)	7%	0	0	yes
18	ada	M	64	0	0	2(2 × 1)	3%	0	0	no
19	pap	M	27	0	4(2 × 2)	4(2 × 2)	--	2	0	no
20	ada	M	32	3(1 × 3)	1(1 × 1)	2(1 × 2)	2%	2	0	no
21	ada	M	57	2(2 × 1)	1(1 × 1)	0	3%	0	2	yes
22	ada	M	53	2(1 × 2)	0	1(1 × 1)	6%	2	0	yes
23	ada	F	43	2(2 × 1)	0	0	1%	3	2	yes
24	ada	F	36	0	--	0	5%	0	3	yes
25	adaprim	M	62	0	0	1(1 × 1)	15%	2	0	yes
26	ada	F	30	0	--	--	--	1	1	yes
27	adaprim	M	10	4(2 × 2)	0	4(2 × 2)	20%	2	2	no
28	ada	M	63	2(2 × 1)	--	--	20%	1	0	no
29	pap	M	49	0	0	2(1 × 2)	15%	2	0	no
30	adarec	F	12	4(2 × 2)	3(1 × 3)	4(2 × 2)	--	0	0	yes
31	ada	F	68	4(2 × 2)	0	6(3 × 2)	10%	1	3	no
32	ada	M	28	1 (1 × 1)	--	--	3%	2	0	yes
33	ada	M	77	0	--	--	2%	3	0	no
34	ada	F	52	0	--	--	4%	3	2	yes
35	adarec	M	12	3(1 × 3)	0	4(2 × 2)	10%	0	1	no
36	ada	M	59	1 (1 × 1)	--	--	18%	2	0	no
37	ada	M	45	2(1 × 2)	--	--	8%	1	0	no
38	pap	M	42	2(1 × 2)	--	--	3%	2	0	yes
39	ada	M	42	4(2 × 2)	--	--	6%	0	1	no
40	ada	F	76	6(3 × 2)	--	--	15%	3	0	no
41	ada	M	39	0	--	--	20%	0	2	no
42	pap	F	50	0	--	--	7%	1	0	yes
43	ada	M	58	6(2 × 3)	--	--	15%	0	1	no
44	adarec	M	21	4(2 × 2)	4(2 × 2)	3(1 × 3)	7%	1	0	yes
45	ada	F	54	2(1 × 2)	--	--	5%	3	1	no

^a^Immunoscore = intensity score x proportion score; ^b^ ada prim is the primitive tumor whose recurrence was not inserted in this table; ada rec and pap rec are tissues from the recurrent tumor, whose primitive was not available. *RT*: radiotherapy

### β-catenin immunostaining score correlated with recurrence

**Fig. 1:**
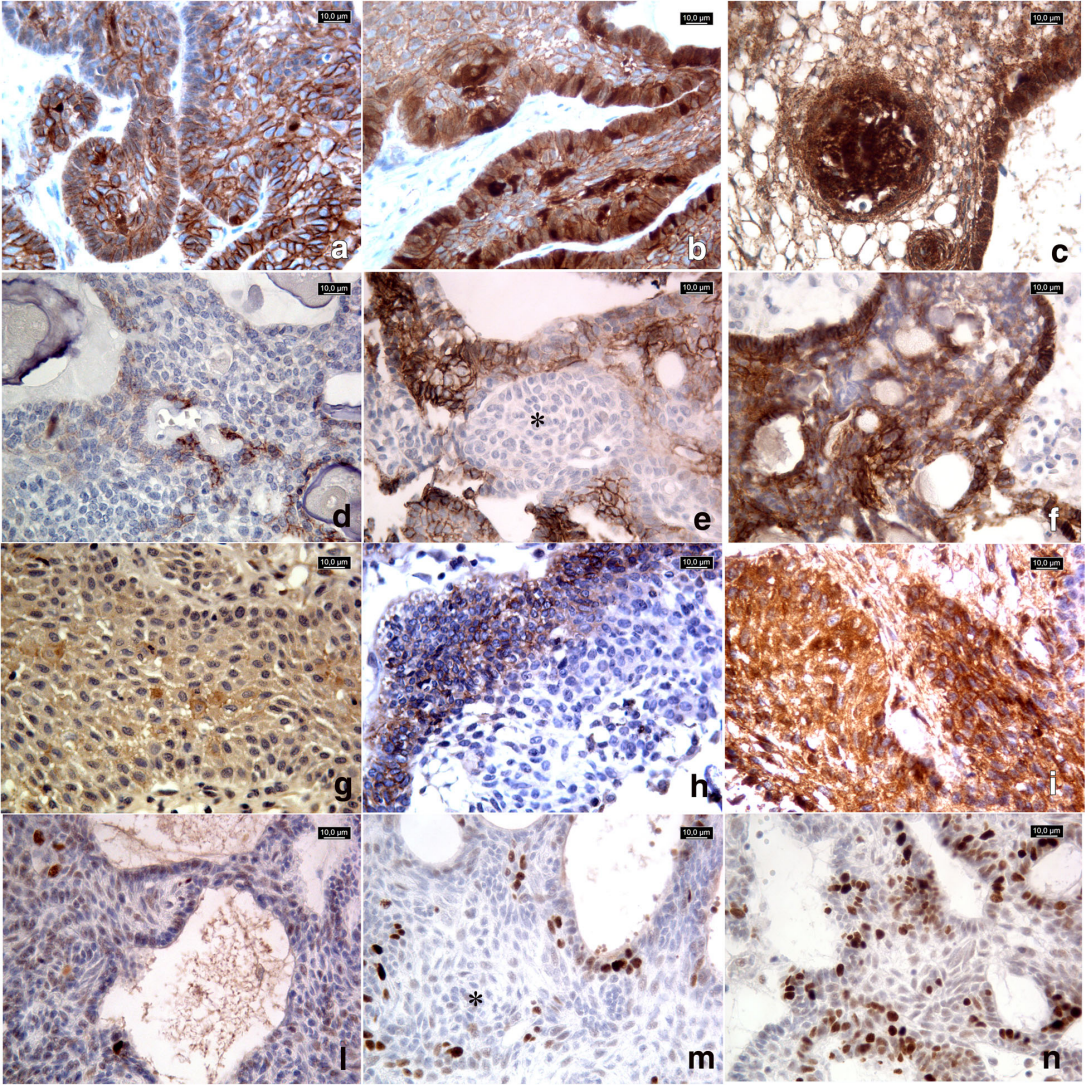
45 cases were examined with β-catenin, Ki67 and pATM antibodies, 30 cases with CD133 and 31 cases with CD166. (**a-c**) β-catenin immunostaining score: moderateand strong nuclear signal in <10% (a, adaCPnr23of Table 1) and in 10–50% (**b**, adaCPnr40) of neoplastic cells, respectively. The signal was more represented in cells forming “whirl-like” structures (**c**, adaCPnr7). **d-f** CD166immunostaining score: strong membranous signal observed in <10% of neoplastic cells (**d**, adaCPnr8); moderate signal observed in 10–50% of cells (**e**, adaCPnr27), sparing the “whirl-like” structures (*). Strong reactivity in >50% of neoplastic cells (**f**, adaCPnr31). **g-i** CD133immunostaining score: signal with moderate intensity observed in 10–50% of neoplastic cells in papCP (**g**, papCPnr14) and in adaCP (**h**, adaCPnr44); strong and diffuse (51–80% of neoplastic cells) immunoreactivity in papCP (**i**, papCPnr13). L-n Ki67 Labeling index**:** low proliferative index Ki67 (<5%) (**l**, adaCPnr3); high Ki67 L.I sparing cells forming “whirl-like” clusters (*) (**m**, adaCPnr15); Ki67 L.I. calculated as 25% (**n**, adaCPnr7). All pictures were captured at 40× magnification

All 45 cases were examined with β-catenin antibody. β-catenin immunoreactivity was detected at the cell membrane in most cases and in the nucleus in 28 cases. We focused on the nuclear signal that was located in neoplastic cells forming “whirl-like” structures and in palisading basal cells arranged around the stellate reticulum. The signal was variable in intensity and proportion of distribution (Fig. 1a–c).

β-catenin immunostaining score was negative in 17 cases and in positive cases it ranged from 1 to 9, being 1, 2, 3, 4, 6 and 9 in 4, 11, 2, 7, 3 and 1 cases, respectively. Based on the calculated IS, all specimens were subsequently divided into two different scoring groups: 32 were absent/low (IS = 0–2) and 13 were moderate/high (IS > 2).

Out of 32 cases with absent/low β-catenin IS, 5 were recurrent, while out of 13 with moderate/high β-catenin IS, 8 were recurrent. Statistical analysis (Table 2) revealed the existence of a significant association between moderate/high β-catenin IS and recurrences in craniopharyngiomas (*p* = 0.0039). No other correlations with clinical variables (age, gender) were found out. A slight tendency to a higher expression of β-catenin was recorded for the adamantinomatoushistotype, rather than the papillary (*p* = 0.0895). In all 7 papCPs that were analyzed, β-catenin-IS was ≤2 (6 cases were 0 and one was 2).

**Table 2:** Examination of correlation between clinical data and immunohistochemical scores

Variables	All cases N=45	B-catenin IS		p value	All cases *N*=30	CD133 IS		*p* value
	(%)	0-2	≥3		(%)	0-2	≥3	
Gender				0.4917				1.0000
Males	30 (67)	20 (62.5)	10 (77)		21 (70)	15 (71)	6 (67)	
Females	15 (33)	12 (37.5)	3 (23)		9 (30)	6 (29)	3 (33)	
Age				0.4113				1.0000
≤18 y	9 (20)	5 (16)	4 (31)		9 (30)	6 (29)	3 (33)	
>18 y	36 (80)	27 (84)	9 (69)		21 (70)	15 (71)	6 (67)	
Histotype				0.0895				0.1432
adaCP	38 (84)	25 (78)	13 (100)		25 (83)	19 (90)	6 (67)	
papCP	7 (16)	7 (22)	0 (0)		5 (17)	2 (10)	3 (33)	
Recurrence				**0.0039**				0.1232
Yes	13 (29)	5 (16)	8 (62)		13 (43)	7 (33)	6 (67)	
No	32 (71)	27 (84)	5 (38)		17 (57)	14 (67)	3 (33)	
Variables	All cases *N*=31	CD166 IS		*p* value	All cases *N*=41	Ki67 L.I.		*p* value
	**(%)**	0-2	≥3		**(%)**	0-2	≥3	
Gender				1.0000				0.4926
Males	21 (68)	11 (65)	10 (71)		29 (71)	12 (63)	17 (77)	
Females	10 (32)	6 (35)	4 (29)		12 (29)	7 (37)	5 (23)	
Age				0.6927				1.0000
≤18 y	9 (29)	4 (31)	5 (36)		7 (17)	3 (16)	4 (18)	
>18 y	22 (71)	13 (69)	9 (64)		34 (83)	16 (84)	18 (82)	
Histotype				0.6358				0.6681
adaCP	26 (84)	15 (88)	11 (79)		35 (85)	17 (89)	18 (81)	
papCP	5 (16)	2 (12)	3 (21)		6 (15)	2 (11)	4 (19)	
Recurrence				**0.0040**				**0.0385**
Yes	13 (42)	3 (76)	10 (71)		11 (27)	2 (10)	9 (41)	
No	18 (58)	14 (24)	4 (29)		30 (73)	17 (90)	13(59)	

All statistcally significant findings were highlighted with bold

Ki67 immunostaining was available in 41 cases and it showed a mean value of 8% (median was 6%, range1–25%). A statistically significant (*p* = 0.0385) association between high Ki67 L.I. (>5%) and recurrences was found but not as strong as with β-catenin IS. Cells forming “whirl-like” clusters did not show high Ki67 L.I (Fig. 1l–n).

### CD166 staining was not equally distributedthroughout the adaCPtumor tissue and its IS was associated with recurrence

CD166 was evaluated on 31 cases. The IS was negative in 7 cases and positive in 24 cases, ranging from 1 to 6 and being respectively 1, 2, 3, 4 and 6 in 5, 5, 4, 8 and 2 cases. 17 cases were assigned to the absent/low (IS = 0–2) scoring group, while 14 to the moderate/high (IS > 2).

The signal was mainly located at the cell membrane (Fig. 1d–f) with diffuse distribution, howeversparing the whirl-like cell clusters, which showed nuclear β-catenin accumulations (Fig. 1e) in adaCPs. In papCPs the immunostainingappeared more homogeneous.

Out of 13 recurrent cases, 3 showed a CD166-IS ≤ 2, while in 10 cases the immunoscore was > 2. Fisher’s exact test showed that CD166-IS was significantlyrelated with recurrence risk (*p* = 0.0040).

CD133 displayed membrane immunostaining with variable distribution (Fig. 1g–i) in both adaCPs and papCPs but its localization was not exclusively identified in cell clusters, as described in literature.

No statistically significant associations between clinicopathologic variables (age, gender, histotype and recurrence) and CD133-IS were observed.

The Spearman test showed direct correlation between β-catenin and Ki67 (*p* = 0.0011, *r* = 0.4903), β-catenin and CD166 (*p* = 0.0002, *r* = 0.6218) and CD166 and Ki67 (*p* = 0.0054, *r* = 0.5119); no significant relation was disclosed between β-catenin and CD133 (*p* = 0.4156, *r* = 0.1543).

### Cytoplasmic pATM was expressed significantly higher in adaCPcompared to papCP

**Fig. 2:**
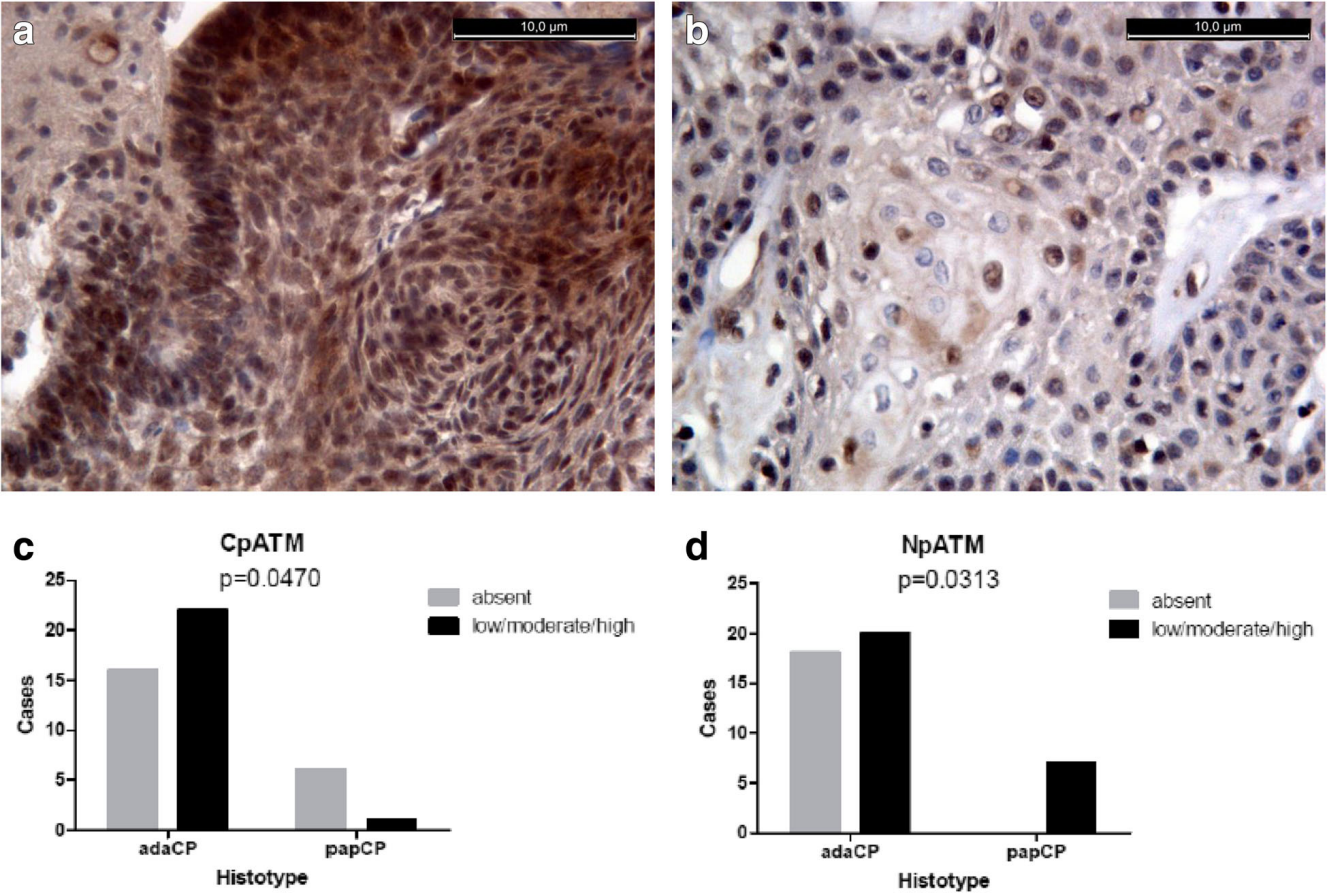
Cytoplasmic signal observed in adamantinomatoushistotype (**a**); mainly nuclear signal in papCPs. CpATM was significantly more expressed in adaCPs (*p* = 0.0470) (**c**), while NpATM was more present in papCPs (*p* = 0.0313) (**d**)

Twenty seven cases out of 45 showed nuclear expression of pATM (Fig. 2b): 9, 11 and 7 cases, each apart respectively with IS of 1, 2 and 3. All papCPs showed nuclear reactivity (7/7).

In 23 out of 45 cases a cytoplasmic reactivity (Fig. 2a) was detected (9, 10 and 4 cases showed 1, 2 and 3 IS respectively). Only 1/7 papCP and 16/28 adaCPs were positive.

We compared both pATM expressions, nuclear and cytoplasmic, in both CP subtypes, by Fisher’s exact test and found out that NpATM was significantly more expressed in papCPs (*p* = 0.0313), while CpATM was more present in adaCPs (*p* = 0.0470) (Fig. 2c, d; Table 2). No differences were found out when we analyzed the different scoring groups (IS 1, 2 or 3).

Finally, we analyzed the likelihood of recurrence for papCPs, on the base of NpATM, and adaCP, on the base of CpATM; no statistically significant correlation was observed (Table 3).

**Table 3:** Examination of correlation between clinical data and immunohistochemical scores

Variables	All cases *N* = 45	NpATM IS		*p* value	All cases *N* = 45	CpATM IS		*p* value
	(%)	0	1–3		(%)	0	1–3	
Gender				0.7477				0.0574
Males	30 (67)	13 (72)	17 (63)		30 (67)	18 (81)	12 (52)	
Females	15 (33)	5 (28)	10 (37)		15 (33)	4 (19)	11 (48)	
Age				0.4487				0.4591
≤18 y	9 (20)	5 (28)	4 (15)		9 (20)	3 (14)	6 (26)	
>18 y	36 (80)	13 (72)	23 (85)		36 (80)	19 (86)	17 (74)	
Histotype				**0.0313**				**0.0470**
adaCP	28 (80)	18 (100)	20 (74)		38 (84)	16 (73)	22 (96)	
papCP	7 (20)	0 (0)	7(26)		7 (16)	6 (27)	1 (4)	
Recurrence^a^				1.0000				1.0000
Yes	1 (14)	0 (0)	1 (17)		12 (32)	8 (32)	4(31)	
No	6 (86)	1 (100)	5 (83)		26 (68)	17 (68)	9 (69)	

^a^Recurrence was evaluated only among papCP for NpATM and only among adaCP for CpATM

All statistcally significant findings were highlighted with bold

## Discussion

The unpredictable features and biological behavior, along with anatomical relationships that craniopharyngiomas establish, represent a key aspect to be considered to rule out prognosis and recurrence risk of these lesions.

β-catenin is a protein with a dual function, regulating cell-cell adhesion and gene transcription. It plays a key role in the Wnt signaling that is involved in morphogenesis and in the process of motility in adaCPs. It is known that exon3 deletion of CTNNB1 gene causes β-catenin intranuclear accumulation that can be highlighted by immunohistochemistry and, though it can represent a diagnostic hallmark.

In our study we examined the nuclear expression of β-catenin in adaCP specimens from 45 patients and investigated the association between the signal and recurrence risk. We found out that a moderate/high signal was more often detected in recurrent tumors.

Probably this could be related to the role of co-transcription factor: indeed, when inside the nucleus, β-catenin binds transcriptional activators, switching on target genes [24].

Furthermore, Wnt signaling and the elevated level of β-catenin are involved in the maintenance of pluripotency in many cell types [25]. Few studies focused the attention on the presence of a stem cell component among CPs (10, 11): some authors supposed that β-catenin accumulating cell clusters could be part of a stem cell niche and may contribute to tumor recurrence. We showed that a medium/high average number of clusters is associated with a higher recurrence rate, whether their sporadic presence is not enough. On the contrary, the relationship with stem cells is not clear: we noted that CD166 correlates with β-catenin and the presence of recurrence, whilst the opposite aspect was seen for CD133.

CD166 is an activated leukocyte cell adhesion molecule (ALCAM), with a controversial role in cancer: immunohistochemical studies on melanocytic lesions showed that CD166 overexpression was associated with tumor progression [26], as well as in bladder cancer with stage/grade [27] and in esophageal squamous cell carcinoma with poor prognosis [28]. In breast cancer, instead, reduced expression of ALCAM was associated with poor prognosis [29]. Moreover, it was shown that human colorectal and prostate cancer stem cells were characterized by the expression of this adhesion molecule [30, 31].

In our series, we observed that CD166 was expressed in CPs and it showed a membrane signal whose immunoscore correlated with β-catenin, Ki67 and recurrence risk. It remains unclear whether it behaves as a stem cell marker in CPs: though, it seems to spare the whirl-like cell clusters in adaCPs, where there should be the stem cell niches. However, regardless of its role in stemness, it can be of help, together with β-catenin, to predict recurrences in CPs. In our cohort CD133 was expressed in both histotypes, while it was described to be almost undetectable in papCPs (11).

Many other markers of stemness (SOX9, SOX2, CD44, NANOG, etc) have been studied in pituitary lesions and, curiously, in adaCP, SOX9 nuclear immunostaining was observed not only in the palisaded basal cell layer of the epithelium, but also in the stellate stromal cells under the epithelium [32]. The same authors also found a correlation between relapse and a high expression level of SOX9. In our series we did not find reactivity of CD133 nor of CD166 in the stellate stromal cells under the epithelium, but only throughout it, just like other markers that had been studied by Chang et al. Therefore, we could hypotize that SOX9 is a marker of progenitor mesenchymal stem cells and represents a more precocius marker of stemness whose higher expression influences the tumor behavior (eg relapse).

The potential of Ki67 as prognostic marker was confirmed. A cut-off of 5% was established as a valid tool to effectively distinguish highly proliferative forms from those with a low rate of proliferation. The Labeling Index was calculated on cells other than those of the basal layer where a high proliferative activity was expected.

Our results could weigh in on the need of a prognostic algorithm for the management of craniopharyngiomas: the identification of a batch of prognostic factors, indeed, could be helpful to determine a more appropriate integrated therapeutic modality.

As well, pATM expression was assessed in the attempt of identifying potential factors that can affect radiosensitivity in CPs. This kinase has a specific role in the DNA double-strand breaks repair, that is exercised in the nucleus, and a cytoplasmic function that is related to autophagy [33], a process that may be implied in therapy resistance [34].

In our series, we observed mainly a nuclear signal in papCPs and a cytoplasmic in adaCs: this allowed us to consider that pATM different expression patterns in CP subtypes might be used as additional diagnostic and prognostic tool. No predictive role of recurrence was observed for pATM, neither cytoplasmic nor nuclear.

The prevalent cytoplasmic localization of pATM in adaCPs may be the effect of a different pathogenetic role of this kinase in the two histotypes. Further studies are needed to clear this aspect.

## Conclusions

In modern histopathology there is a strong need to identify prognostic markers able to predict at best the biology of tumors. In craniopharyngiomas recurrence is an event that should be managed and prevented with multidisciplinary approach, in order to define the most appropriate treatment strategy. Overall, our results demonstrate how β-catenin, CD166 and Ki67 could be a valid supplementary tool. They can be also regarded as potentially useful antitumor targets.

Furthermore, our findings could suggest to introduce pATM as additional distinction-marker between adamantinomatous and papillary histotypes.

## Abbreviations

adaCP: Adamantinomatous craniopharyngiomas
ALCAM: Activated leukocyte cell adhesion molecule
CD133: Cluster of differentiation 133
CD166: Cluster of differentiation 166
CpATM: Cytoplasmic phospho-ATM
CTNNB1: Catenin Beta 1
IS: Immunostaining score
L.I: Labeling index
NpATM: Nuclear pATM
papCP: Papillary craniopharyngiomas
p-ATM: Phosphorilated- Ataxia-Telangiectasia mutated
WHO: World Health Organization
WNT: Wingless type
